# Alternatives to Three Designs for Broadband Sound Absorption

**DOI:** 10.3390/ma19132763

**Published:** 2026-06-30

**Authors:** Keith Attenborough

**Affiliations:** School of Engineering and Innovation, The Open University, Milton Keynes MK7 6AA, Buckinghamshire, UK; keith.attenborough@open.ac.uk

**Keywords:** sound absorbers, metamaterials, partitions, porous materials, sustainability

## Abstract

Three designs for broadband sound absorption by thin hard-backed layers, respectively, combine synthetic porous materials and resonant cavities, embed opposed arrays of horizontal plates in a porous layer and mount multiple parallel microperforated plates in a conical shell. The measured and predicted normal-incidence absorption spectra from these designs are compared with predictions for hard-backed porous layers of the same thickness that either have a small number of embedded vertical and horizontal solid partitions or consist of materials derived from natural sources. The comparisons suggest that broadband sound absorption spectra comparable with those of the more complicated designs can be obtained in ways that are simpler and potentially more sustainable for manufacturing and disposal.

## 1. Introduction

Ideally, sound-absorbing treatments for use in aircraft and vehicle structures, white goods and prefabricated building constructions should provide broadband absorption over the audio-frequency range, while adding as little volume and weight as possible. Typical sound absorption coefficient spectra provided by thin hard-backed layers of synthetic porous materials such as glass wool or polymer foams are poor at low frequencies unless the layer thickness is increased or the layer is mounted on an air gap, thereby adding volume. Designs suitable for additive manufacturing (3D printing) obtain useful subwavelength (i.e., low frequency) absorption from thin layers by incorporating various combinations of resonators, labyrinthine channels (sometimes called ‘space coiling’), microperforated plates and porous materials. Some of these designs are in References [1,2,3,4,5] but these cite references to many others. Other designs involve solid inclusions in a hard-backed porous layer [6,7,8,9,10]. Among these are designs that use vertical and horizontal partitions [6,7], the manufacturing of which through 3D printing should be relatively easy. These partition designs have been validated numerically, and the physical mechanisms they invoke, including local resonance and slow waves, have been employed and verified experimentally many times. But partitioned porous layer ideas are relatively unexplored and there are few independent measurements that support them.

Another approach concerns the choice of porous materials employed for the purpose of broadband sound absorption. Concerns with environmental impact and sustainability have led to increasing interest in developing sound-absorbing materials from natural sources [11,12]. This paper investigates the extent to which sound absorption comparable with or superior to that of three designs [1,2,5] could be achieved with simpler partition-based designs or by using naturally sourced instead of synthetic porous materials.

**Figure 1:**
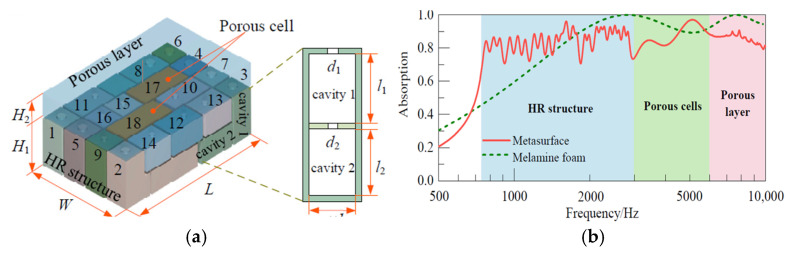
(**a**) A sound-absorbing structure in which L=68 mm, W=44 mm, $H_{1}=21$ mm, $H_{2}=10$ mm. A 10 mm thick melamine foam layer covers eighteen coupled resonator cavities with $l_{1}=26$ mm, $l_{2}=28$ mm, $d_{1}=4.8,\;3.3\;and\;4.5$ mm, $d_{2}=2.3\;and\;1.7$ mm. (**b**) The predicted normal-incidence absorption spectrum for the array (solid red line), with shaded areas indicating contributions from different parts of the structure. Also shown in Figure 1b is a prediction for a 31 mm thick hard-backed melamine foam layer (broken green line). [Adapted from Figure 1 of Liu et al. [1] under the Creative Commons Attribution license version CC by 4.0 https://creativecommons.org/licenses/by/4.0/ (accessed on 17 June 2026)].

The first of the designs to be considered is shown in Figure 1a [1]. A 10 mm thick, melamine foam layer covers an array of eighteen rectangular cavities. These include eight single cavities (numbered 1–8 in Figure 1a) and eight double cavities, i.e., two connected single cavities (numbered 9–16 in Figure 1a). Sound enters the cavities through small holes of various widths (1.7 mm, 2.2 mm and 4 mm), so they act as Helmholtz resonators. The double cavities are folded beneath the single cavities to reduce the total height (31 mm) of the structure. Cavities 17 and 18 in Figure 1a, which are 25 mm long and 10 mm wide, contain 20 mm and 12 mm thick layers of another foam, henceforth called ‘cavity’ foam [1]. Figure 1b shows the frequency ranges encompassing the main contributions to the overall absorption coefficient spectrum from the cavity resonators (HR), cavities with porous infill and the thin, porous covering. Impedance tube measurements were made between 600 Hz and 1600 Hz on a 3D printed replica of the Figure 1a structure. Analytical and numerical predictions were found to agree with the data from these measurements [1].

**Figure 2:**
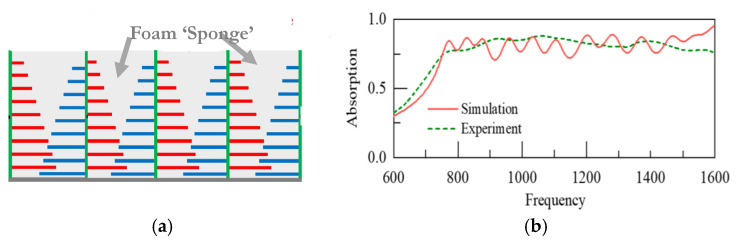
(**a**) An array of unit cells, each of which contains two arrays of nine embedded plates with lengths that increase with depth within a 50 mm thick layer of porous material; (**b**) measured and predicted absorption coefficient spectra [adapted from Figures 1h and 2a in Gao et al. [2] under the Creative Commons Attribution license version CC BY 4.0, https://creativecommons.org/licenses/by/4.0/ (accessed on 17 June 2026)].

Figure 2a shows the second design for broadband sound absorption considered [2].

Unit cells are filled with a porous material containing left- and right-hand series of regularly spaced embedded horizontal plates with lengths that increase with depth. Figure 2b shows a measured normal incidence absorption coefficient spectrum together with a corresponding numerical prediction (labelled simulation).

The acoustical properties of the melamine foam layer, the ‘cavity’ foam used in the Figure 1a design [1] and the foam ‘sponge’ used in the Figure 2a design [2], are calculated using the Johnson–Champoux–Allard (JCA) model for a rigid-framed porous material. Expressions for the bulk complex density $\rho_{1}\left(\omega \right)$ and compressibility $C_{1}\left(\omega \right)$ are [13]

(1) $$\rho_{1}\left(\omega \right)=\frac{\alpha_{\infty }\rho_{0}}{\phi }\left[1+\frac{i\sigma \phi }{\omega \rho_{0}\alpha_{\infty }}G(\Lambda )\right],$$

(2) $$C_{1}\left(\omega \right)={\phi \left(\gamma P_{0}\right)}^{-1}\left[\gamma -\left(\gamma -1\right){\left[1+\frac{i\sigma \phi }{\omega \rho_{0}\alpha_{\infty }N_{Pr}}G^{\prime }\left(\Lambda^{\prime }\right)\right]}^{-1}\right]$$

(3) $$G\left(\Lambda \right)=\sqrt{\left(1+\frac{4i{\alpha_{\infty }}^{2}\eta \omega \rho_{0}}{{\left(\sigma_{\rho }\Lambda \phi \right)}^{2}}\right)}$$

(4) $$G^{\prime }\left(\Lambda^{\prime }\right)=\sqrt{\left(1+\frac{4i{\alpha_{\infty }}^{2}\eta N_{pr}\omega \rho_{0}}{{\left(\sigma_{C}\Lambda^{\prime }\phi \right)}^{2}}\right)}$$

where the $e^{-i\omega t}$ time convention is understood, ω=2πf (rad s^−1^) is the angular frequency, f (Hz) is the frequency, t (s) is time, $\alpha_{\infty }$ is tortuosity, ϕ is porosity, σ is flow resistivity with units of Pa s m^−2^, $\rho_{0}$ is the density of air (kg m^−3^). In Equations (3) and (4), $\sigma_{\rho }=\frac{8\mu \alpha_{\infty }}{\phi \Lambda^{2}}$ and $\sigma_{C}=\frac{8\mu \alpha_{\infty }}{\phi {\Lambda^{\prime }}^{2}}$, where Λ is a characteristic viscous length and $\Lambda^{\prime }$ is a characteristic thermal length. $N_{pr}$ and η are the Prandtl number and dynamic viscosity coefficient for air, respectively.

The characteristic impedance, $Z_{1}$, and the propagation constant, $k_{1}$, are calculated, respectively, from $\rho_{1}\left(\omega \right)$ and $C_{1}\left(\omega \right)$ using

(5) $$Z_{1}=\sqrt{\rho_{1}\left(\omega \right)/C_{1}\left(\omega \right)}$$

(6) $$k_{1}=\omega \sqrt{\rho_{b}\left(\omega \right)C_{b}\left(\omega \right)}$$

The specific surface impedance, $Z\left(d\right)$, of a hard-backed porous layer of thickness d is given by Equation (7)

(7) $$Z\left(d\right)=Z_{1}coth\left(-ikd\right)$$

The plane wave reflection coefficient, *R*(*d*), and the normal-incidence absorption coefficient, α(*d*), for a hard-backed porous layer of thickness *d* are given by Equations (8) and (9), respectively.

(8) $$R\left(d\right)=\frac{1-Z\left(d\right)}{1+Z\left(d\right)},$$

(9) $$\alpha \left(d\right)=1-\left|{\left(R\left(d\right)\right)}^{2}\right|,$$

The JCA parameter values used to calculate the acoustic properties of the three porous materials used in the first two structures [1,2] are listed in Table 1.

**Figure 3:**
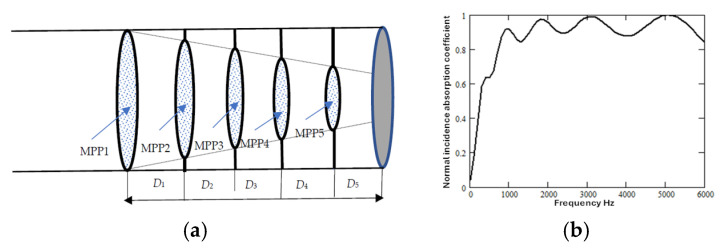
(**a**) A generalized schematic of an array of five microperforated plates (MPP) in a conical shell; (**b**) the absorption coefficient spectrum predicted for five MPPs containing holes with diameter 0.2 mm and depth 0.2 mm, perforation ratio 4%, equally spaced at 12 mm inside a conical tube with a radius that decreases linearly from 30 mm to 1 mm at the back wall [adapted from Figures 7 and 8 in Ortiz et al. [5] under the Creative Commons Attribution license version CC BY 4.0, https://creativecommons.org/licenses/by/4.0/ (accessed on 17 June 2026)].

Figure 3a shows the third example of a broadband sound-absorbing design to be considered. It is based on the sonic black hole concept. It consists of parallel microperforated plates inside a conical shell [4]. Figure 3b shows the numerically predicted absorption coefficient spectrum for an arrangement of five MPP equally spaced at 12 mm inside a conical tube with a radius that decreases linearly from 30 mm to 1 mm at the back wall.

To investigate the extent to which sound absorption coefficient spectra comparable with or superior to those obtained with the designs in Figure 1a, Figure 2a and Figure 3a could be achieved in simpler or more sustainable ways, it is useful to introduce a Figure of Merit (FOM) [3] defined in Equation (10).

(10) $$FOM=\alpha_{avg}\left(\alpha_{target}\right)\times \frac{∆f}{f_{r}}\times \frac{\lambda_{max}}{L},$$

where $\alpha_{avg}$ is the average absorption coefficient above the target value ($\alpha_{target}$), ∆f is the frequency range in which $\alpha >\alpha_{target}$, $f_{r}$ is the overall frequency range of interest, $\lambda_{max}$ is the wavelength at the lowest frequency at which the target absorption coefficient value is reached, and L is the total thickness of the metamaterial absorber. FOM not only indicates the bandwidth above the target absorption but also accounts for the lowest frequency at which the target absorption is reached. The definition in Equation (10) differs from that used elsewhere [3] since it uses thickness, L, rather than the cube root of volume. Although the overall volume is important to potential applications, the use of thickness is more relevant for the later comparisons with the absorption performance of simple hard-backed porous layers. Also, rather than $\alpha_{target}$ = 0.7 as in [3], $\alpha_{target}$ is set at 0.8 for the FOM calculations in this paper, which should be sufficient for most practical applications.

Table 2 summarizes the values used in calculating FOM for the three designs in Figure 1a, Figure 2a and Figure 3a. Those for the Figure 1a design use the numerically predicted wideband absorption performance rather than the data which are limited to an upper frequency of 1.6 kHz. Similarly, those for the Figure 3a design are based on the predicted performance shown in Figure 3b since no data are available [5]. On the other hand, the FOM for the design in Figure 2a is calculated from data [2].

A simpler and relatively unexplored alternative to the designs in Figure 1, Figure 2 and Figure 3 consists of thin solid vertical and horizontal partitions embedded in hard-backed layers of a porous material [6]. Another alternative to be considered is a hard-backed layer of a porous material with higher tortuosity than those used in the designs in Figure 1a and Figure 2a. Section 2 outlines theory for the acoustical properties of partitioned structures. Section 3 compares predictions of normal-incidence sound absorption coefficient for partitioned porous layers with data and predictions for the structures shown in Figure 1a, Figure 2a and Figure 3a. Section 4 contains predictions based on the use of alternative (unpartitioned) porous materials with higher tortuosity and Section 5 offers concluding remarks.

## 2. Theory of Partitioned Porous Layers

**Figure 4:**
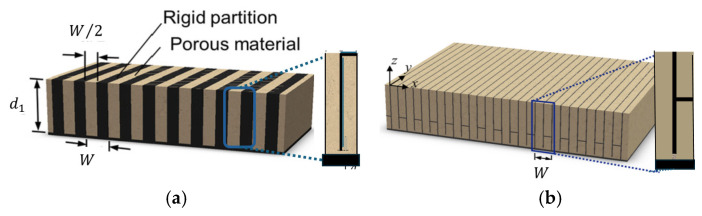
Arrays of adjacent unit cells containing (**a**) type 1 and (**b**) type 2 partitions in a porous material. Each cell contains vertical and horizontal solid partitions, creating regions connected by the gap below the vertical partition.

Hard-backed layers of porous material containing adjacent unit cells containing partitions of types 1 and 2 are illustrated in Figure 4.

**Figure 5:**
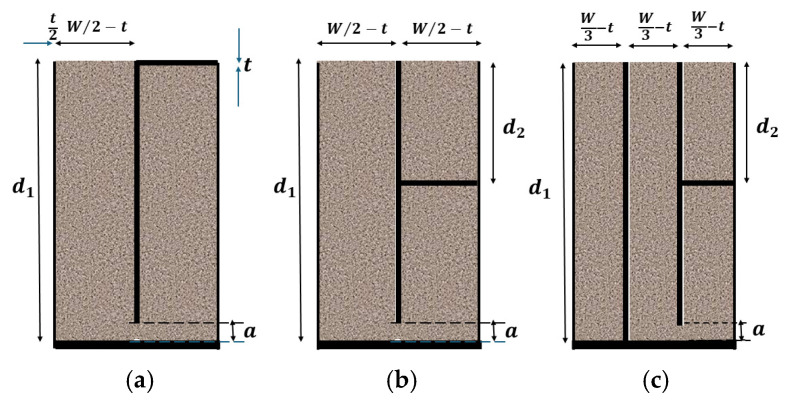
Dimensions of unit cells of types (**a**) 1, (**b**) 2 and (**c**) 3.

A third type of partition and the dimensions associated with all three types of partitions are shown in Figure 5.

**Figure 6:**
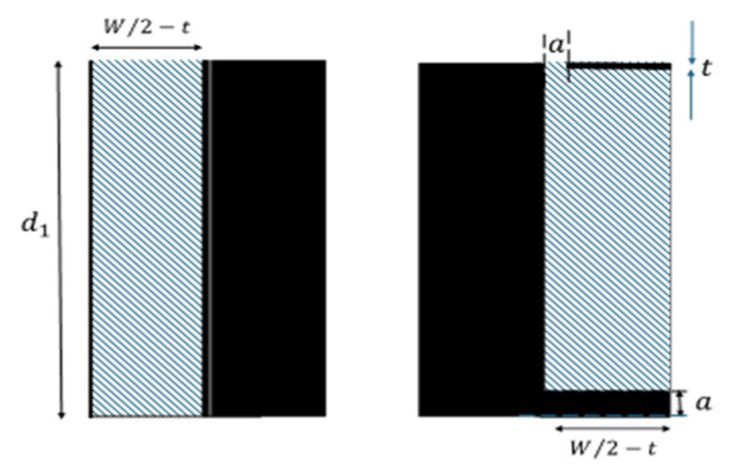
Sub-cells of unit cell type 1 [4].

Using an effective medium approach, the overall impedance of unit cell type 1 (Figure 5a) is calculated by considering the two sub-cells shown in Figure 6 [6].

The left-hand sub-cell of width $\left(W/2-t\right)$, where *W* is the total width of the unit cell and *t* is the thickness of the partition, has the characteristic impedance $Z_{A}^{c}$ given by [6]

(11) $$Z_{A}^{c}=\rho_{0}c_{0}\frac{Z_{1}}{\eta_{1}},$$

where $Z_{1}$ is given by Equation (8).

The approximate impedance, $Z_{B}$, of the right-hand sub-cell of width $\left(W/2-t\right)$ and length $\left(d_{1}-a-t\right)$, is given by [6]

(12) $$Z_{B}\approx \frac{-i}{\eta_{1}}\left[\omega m-Z_{A}^{c}\cot\left(k_{1}\left(d_{1}-t-a\right)\right)\right],$$

(13) $$m=\left(t+2\delta a\right)\rho_{1\;}\frac{W}{2a},$$

(14) $$\eta_{1}=\left(W/2-t\right)/W,$$

where δ = 1.9, *m* is the acoustic mass of the gap and a is the width of the gap. The gap is considered to form the neck of a resonator filled with porous material [6]. The approximation in Equation (12) relies on the gap being sufficiently small that differences from standard radiation conditions are not important.

The specific surface impedance, $Z_{AB}$, of a unit cell with a type 1 partition is given by [6]

(15) $$Z_{AB}=\frac{Z_{A}^{c}}{\rho_{0}c_{0}}\left[\frac{Z_{B}\cot\left(k_{1}d_{1}\right)-iZ_{A}^{c}}{-iZ_{B}+Z_{A}^{c}\cot\left(k_{1}d_{1}\right)}\right].$$

The reflection and absorption coefficients are obtained by replacing $Z\left(d\right)$ by $Z_{AB}$ in Equations (8) and (9) respectively.

**Figure 7:**
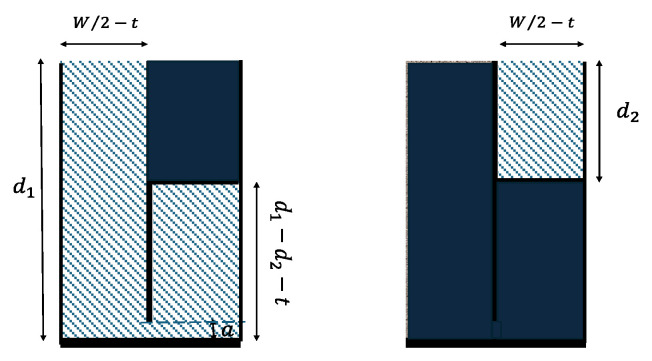
Sub-cells of unit cell type 2 [4,5].

Figure 7 shows the sub-cells for type 2 partitions [6]. The left-hand part of the sub-cell is connected to the resonant absorbing volume of length $\left(d_{1}-d_{2}-t\right)$ and width $\left(W-t\right)/2$ in the right-hand half of the unit cell by the gap of width a at the bottom of the vertical partition.

If, as for the type 1 arrangement, the gap is treated as the neck of a Helmholtz resonator, the specific surface impedance, ${Z_{AB}}^{\prime }$, for this sub-cell is given by Equation (16)

(16) $${Z_{AB}}^{\prime }=\frac{Z_{A}^{c}}{\rho_{0}c_{0}}\left[\frac{{Z_{B}}^{\prime }\cot\left(k_{1}d_{1}\right)-iZ_{A}^{c}}{-i{Z_{B}}^{\prime }+Z_{A}^{c}\cot\left(k_{1}d_{1}\right)}\right],$$

(17) $${Z_{B}}^{\prime }\approx \frac{-i}{\eta_{1}}\left(\omega m-\rho_{0}c_{0}Z_{1}\cot\left(k_{1}\left(d_{1}-d_{2}-t\right)\right)\right),$$

The specific surface impedance, $Z_{C}$, of the right-hand sub-cell is given by

(18) $$Z_{C}=\left(Z_{1}/\eta_{1}\right)\cot\left(k_{1}d_{2}\right),$$

Hence, the specific surface impedance of a unit cell with type 2 partitions is given by

(19) $${\left(Z_{type2}\right)}^{-1}={\left({Z_{AB}}^{\prime }\right)}^{-1}+{\left(Z_{c}\right)}^{-1},$$

The specific surface impedance of cell type 3 is the sum of the admittances of a sub-cell of type 2 and that of a sub-cell of a hard-backed porous layer of length $d_{1}$ corresponding to the left-hand sub-cell in Figure 6 after allowing that each sub-cell has a narrower width (W/3−t).

(20) $${\left(Z_{type3}\right)}^{-1}={\left({Z_{type2}}^{\prime }\right)}^{-1}+{\left(Z_{D}\right)}^{-1},$$

(21) $$Z_{D}=\left(Z_{1}/\eta_{2}\right)\cot\left(k_{1}d_{1}\right),$$

(22) $$\eta_{2}=\left(W/3-t\right)/W,$$

(23) $$m^{\prime }=\left(t+2\delta a\right)\rho_{1\;}W/\left(3a\right).$$

${Z_{type2}}^{\prime }$ in Equation (20) is calculated from Equations (16)–(19) after replacing $\eta_{1}$ by $\eta_{2}$ and m by $m^{\prime }$.

## 3. Predictions and Comparisons with Data

### 3.1. Predictions for Three Partition Types

**Figure 8:**
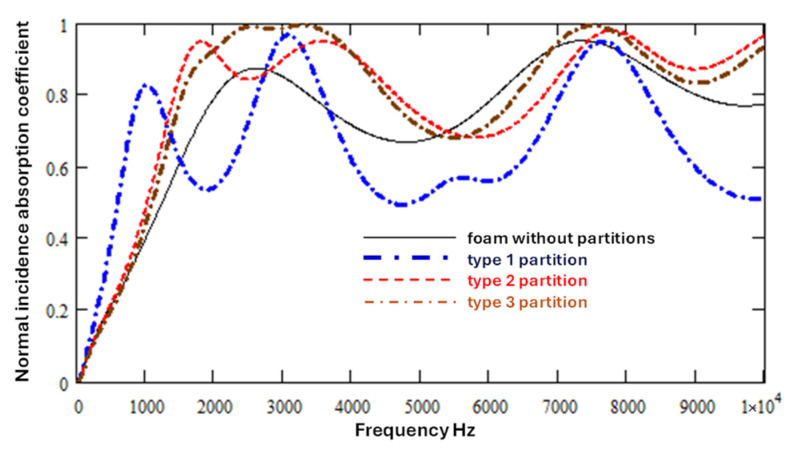
Normal-incidence absorption coefficient spectra predicted for three types of partition and for the unpartitioned porous material (porosity 0.95, tortuosity 1.42, viscous characteristic length 180 μm, thermal characteristic length 360 μm and flow resistivity 8.9 kPa s m^−2^) with $d_{1}=30$ mm, $d_{2}=21$ mm, a=1 mm and t=0.5 mm.

To represent the acoustical properties of the porous foam in which partitions are embedded, Yang et al. [6] use the JCA model with porosity 0.95, tortuosity 1.42, viscous characteristic length 180 μm, thermal characteristic length 360 μm and flow resistivity 8.9 kPa s m^−2^. Figure 8 shows normal-incidence absorption coefficient spectra predicted for this foam containing each of the three types of partitions with $d_{1}=30$ mm, $d_{2}=21$ mm, a=1 mm and t=0.5 mm.

Also shown is the prediction for the 30 mm thick layer of foam without partitions.

**Figure 9:**
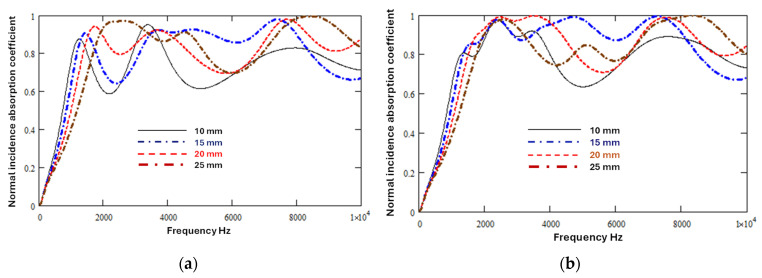
Predicted influence of $d_{2}$ on normal-incidence sound absorption coefficient spectra for (**a**) type 2 and (**b**) type 3 partitions in foam characterized by the JCA model (porosity 0.95, tortuosity 1.42, viscous characteristic length 180 μm, thermal characteristic length 360 μm and flow resistivity 8.9 kPa s m^−2^).

Type 1 partitions are predicted to result in the highest absorption below 1 kHz, but otherwise the resulting absorption spectrum is highly oscillatory. Although predictions for type 2 and type 3 partitions do not show as much absorption as type 1 partitions below 1 kHz, they indicate higher absorption between 500 Hz and 5 kHz than predicted for the foam layer without partitions. The predicted normal-incidence absorption coefficient spectra for 30 mm thick layers of foam [5] with type 2 and type 3 partitions are sensitive to the value of $d_{2}$. This is shown for 10 mm $\leq d_{2}\leq$ 25 mm and type 2 and type 3 partitions respectively in Figure 9a,b.

### 3.2. Absorption Spectra for the Figure 1a Design and Partitioned Porous Material Alternatives

**Figure 10:**
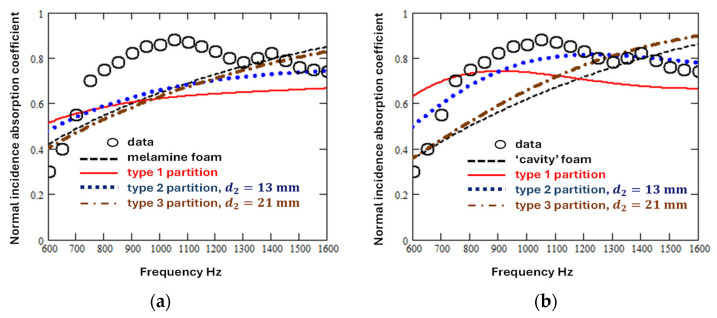
Normal-incidence absorption coefficient data (open circles) for the design in Figure 1a [1] compared with predictions for type 1 partition structure (solid red lines) with $d_{1}=31$ mm a=1 mm and t=0.5 mm, type 2 partition (blue dotted lines; $d_{2}=13$ mm) and type 3 partition structure (brown dash-dot lines; $d_{2}=21$ mm) in (**a**) melamine foam and (**b**) ‘cavity’ foam (see Table 1). Also shown (broken black lines) are predictions for the foams without partitions. The data in Figure 10a were obtained using ChatGPT (version 5.5, OpenAI) to read the data corresponding to the curve ‘experiment’ from an image of Figure 8b in [1].

Figure 10a,b show data from measurements of normal-incidence absorption spectra made by Liu et al. [1] on a 3D-printed version of the structure in Figure 1a over the frequency range from 600 Hz to 1600 Hz.

These data are compared with predictions for three types of partitions in each foam with the parameters in Table 1 and with predictions for 31 mm thick foam layers without partitions. The absorption predicted for a 31 mm thick layer of the ‘cavity’ foam is lower than the same thickness of melamine foam since it has a lower flow resistivity. But, with type 2 partitions, the ‘cavity’ foam is predicted to yield an absorption spectrum comparable to that measured for the Figure 1a structure between 700 Hz and 1200 Hz. The insertion of partitions in the ‘cavity’ foam is predicted to improve absorption spectra more than predicted if the melamine foam has the same partitions.

**Figure 11:**
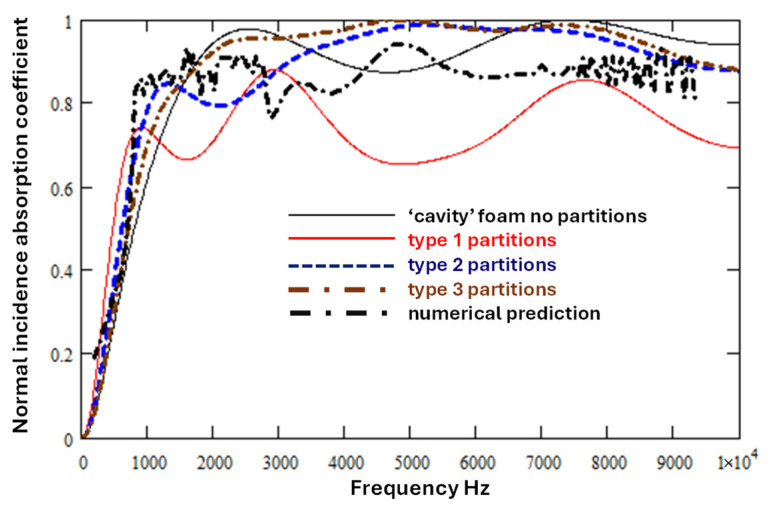
Normal-incidence absorption coefficient spectra predicted for three types of partitions (type 1—solid red line; type 2—broken blue line and type 3—dash dot brown line, $d_{1}=31$ mm, $d_{2}=15$ mm, a=1 mm and t=0.5 mm) in a 31 mm thick hard-backed layer of ‘cavity’ foam [1] (see Table 1) compared with numerical predictions (dash dot black line) for the design shown in Figure 1a. The numerical prediction curve was obtained by using ChatGPT to give points on the curve labeled ‘metasurface’ from an image of Figure 6b in [1].

Figure 11 compares predictions of absorption coefficient spectra up to 10 kHz for the three types of partitions in the ‘cavity’ foam ($d_{1}=31$ mm, $d_{2}=15$ mm, a=1 mm and t=0.5 mm), the same thickness of ‘cavity’ foam without partitions and the numerical prediction for the structure in Figure 1a [1].

A type 1 partition in the ‘cavity’ foam [1] is predicted to give higher absorption than predicted for the Figure 1a structure below 500 Hz but less at higher frequencies. However, type 2 partitions in the ‘cavity’ foam are predicted to match the numerical prediction of absorption for the Figure 1a structure below 700 Hz, giving less absorption than the numerical predictions between 1 and 2 kHz but higher absorption above 3 kHz. The predicted absorption coefficient spectrum for type 3 partitioning is less than the numerical prediction for the Figure 1a structure between 500 Hz and 1.5 kHz but consistently higher thereafter. Moreover, the prediction for type 3 partitions is higher than predicted for the ‘cavity’ foam without partitions between 3 and 6 kHz. Overall, the predicted broadband absorption spectra with type 2 and type 3 partitions in the ‘cavity’ foam are comparable with that predicted for the design in Figure 1a.

This observation is supported by the FOM values predicted for the insertion of type 2 and type 3 partitions in the ‘cavity’ foam listed in Table 3, which are comparable with those calculated from the numerical prediction for the Figure 1a structure (10.992).

### 3.3. Absorption Spectra for the Figure 2a Design and Partitioned Porous Material Alternatives

**Figure 12:**
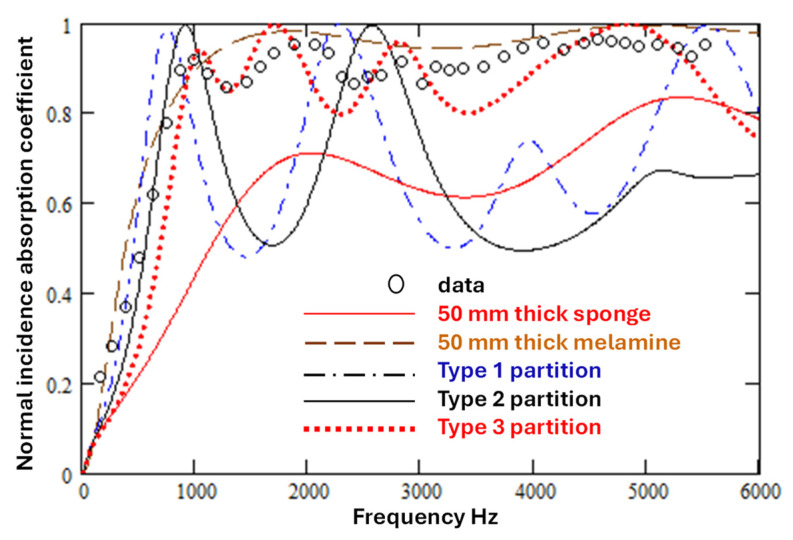
Normal-incidence absorption coefficient data for the Figure 2a design [2] and predictions for 50 mm thick ‘sponge’, melamine foam and for ‘sponge’ containing each of three types of partition ($d_{1}=50$ mm, a=1 mm, t=0.5 mm, $d_{2}=15$ mm (type 2) and 21 mm (type 3)). JCA parameters for the materials are listed in Table 1. The data points were obtained by using ChatGPT to read the points labeled ‘Test’ from an image of Figure 19d in [2].

The Figure 2a design [2] represents an extension of that proposed by Yang et al. [8], who consider only the ‘right-hand’ arrays of the horizontal partitions shown in Figure 2a. Figure 12 compares the normal-incidence absorption coefficient spectrum for the Figure 2a design measured on a 3D printed sample [2] with predictions for unpartitioned 50 mm thick ‘sponge’ and melamine foam layers and for a 50 mm thick layer of the ‘sponge’ material containing the three types of partition (Figure 4).

The porous foam ‘sponge’ used in the Figure 2a design [2] has relatively low flow resistivity (see Table 1). Consequently, the absorption spectrum predicted for a 50 mm hard-backed layer of this material is relatively low (solid red line in Figure 12). Nevertheless, insertion of type 3 partitions in the ‘sponge’ is predicted to give a comparable absorption spectrum to that achieved with the multiple horizontal embedded plate Figure 2a design. Moreover, the normal-incidence absorption spectrum for a 50 mm thick hard-backed (unpartitioned) melamine foam with the JCA parameter values listed in Table 1 (broken brown line in Figure 12), matches or exceeds that measured for the design in Figure 2a with ‘sponge’ infill. These observations are supported by the associated FOM values listed in Table 4, which are comparable with those calculated for the Figure 2a structure (6.340).

### 3.4. Comparison of Predictions for the Design in Figure 3a and Partitioned ‘Cavity’Foam

**Figure 13:**
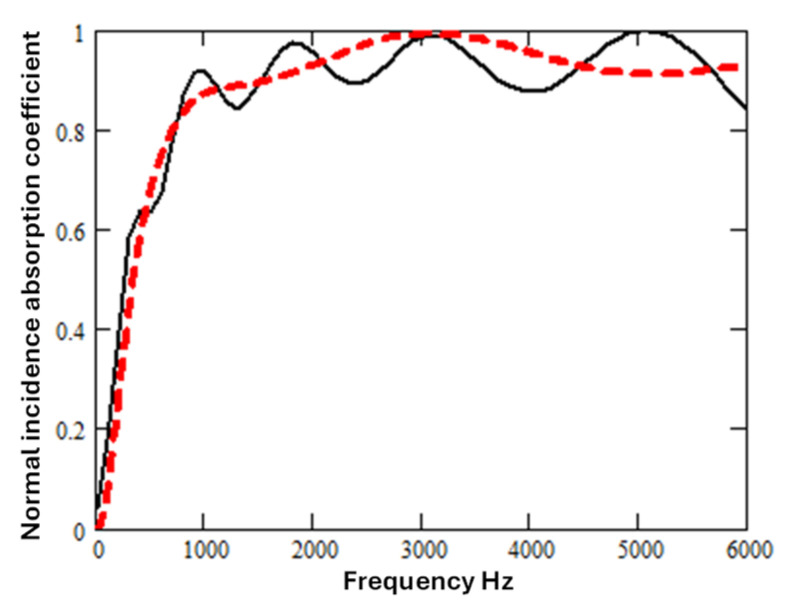
Comparison of the normal-incidence absorption coefficient spectrum predicted for the 60 mm thick Figure 3a design (solid black line) [5] with that predicted for the same thickness of ‘cavity foam (see Table 1) containing type 3 partitions (broken red line, $d_{2}=25$ mm). The curve of the prediction for the Figure 3a design was obtained by using ChatGPT to read the corresponding (blue) curve in an image of Figure 8 in [5].

Figure 13 compares the absorption spectrum predicted for the 60 mm thick Figure 3a design with that predicted for the same thickness of the ‘cavity’ foam used in the Figure 1a design with type 3 partitions ($d_{2}=25$ mm). Use of type 3 partitions in the ‘cavity’ foam layer is predicted to offer a comparable absorption spectrum to that predicted for the microperforated plate array. Indeed, not only does the predicted spectrum for type 3 partitions have fewer oscillations, but also it is so similar that it is superfluous to investigate the associated FOM, which will be similar to that for the Figure 3a design in Table 1.

## 4. Predictions for Naturally Sourced Materials

The low-frequency absorption of a thin hard-backed layer of a rigid porous material is improved by increasing the tortuosity of the material [9,10]. Naturally sourced porous materials tend to have higher tortuosity values than conventional synthetic materials [10] as well as having less environmental impact with respect to manufacturing and disposal. Impedance tube measurements have been made on various naturally sourced samples, and values of the JCA model parameters, other than flow resistivity and porosity, which were measured independently, have been obtained by fitting the impedance tube data. Parameters for 30 mm and 50 mm thick samples made from sugarcane bagasse and Typha fiber (with bulk density 200 kg m^−3^) are listed in Table 5 [11,12].

**Figure 14:**
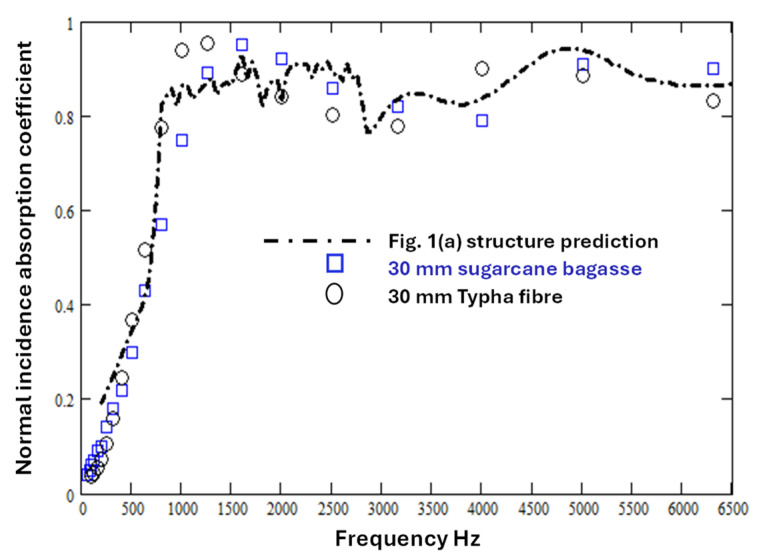
Normal-incidence absorption coefficient data for 30 mm thick layers made from sugar cane bagasse (☐) and Typha fiber (O) compared with the numerical prediction (dash dot black line) for the design shown in Figure 1a [1]. The curve corresponding to the prediction for the Figure 1a structure was obtained as described in the caption for Figure 11.

The JCA-fitted tortuosity values for these materials, i.e., between 1.5 and 2.8, are higher than those for the porous foams used in the Figure 1a design, for which the highest fitted tortuosity value is 1.07 (see Table 1). Figure 14 compares normal-incidence absorption coefficient data [10] for 30 mm thick layers made from sugar cane bagasse and Typha fiber with the numerical prediction for the 31 mm thick structure shown in Figure 1a. Below 1.5 kHz the Typha sample offers superior absorption to that of the Figure 1a design. Above 1.5 kHz, the spectra for both Typha fiber and sugarcane bagasse are comparable.

The respective FOM shown in Table 6 supplement these observations. Since its measured absorption coefficient is not 0.8 until approximately 1 kHz, the FOM calculated for the sugarcane bagasse sample is less than predicted for the Figure 1a design (10.992). However, the FOM value for the Typha fiber sample exceeds the FOM for the Figure 1a design significantly.

**Figure 15:**
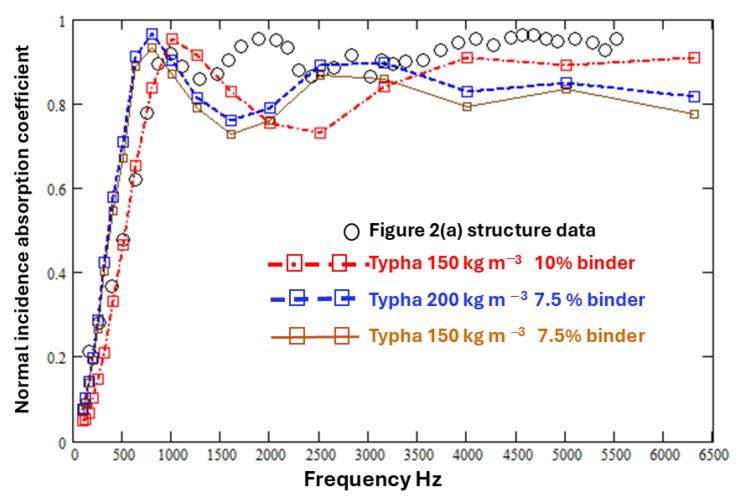
Measured normal-incidence absorption-coefficient spectrum for the Figure 2a structure (O) and for 50 mm thick hard-backed Typha fiber samples with the properties listed in Table 2 and in the key. The data points for the Figure 2a structure were obtained as described in the caption for Figure 12.

Figure 15 compares normal-incidence absorption coefficient data for 50 mm thick layers made from Typha fiber [11] with the numerical prediction for the 50 mm thick structure shown in Figure 2a. The measured absorption coefficient values of the Typha fiber samples above 1 kHz are between 0.8 and 0.85, slightly lower than the measured absorption spectrum of the Figure 2a design. However, the measured absorption coefficient values for the 7.5% binder Typha fiber samples are higher than those measured for the Figure 2a design below 1 kHz.

The comparative usefulness of the naturally sourced samples is indicated by the calculated FOM values listed in Table 7.

**Figure 16:**
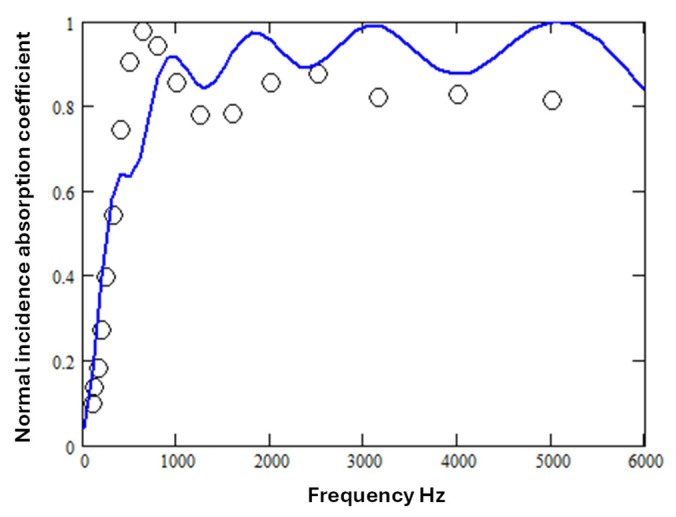
Predicted normal-incidence absorption-coefficient spectrum for the Figure 3a structure (solid blue line) and data (O) for a 60 mm thick hard-backed Typha fiber sample with the properties listed in Table 8 [data supplied by Mohammad SheikhMozafari, Department of Occupational Health Engineering, School of Public Health, Tehran University of Medical Sciences, Tehran, Iran]. The curve of the prediction for the Figure 3a structure was obtained as described in the caption for Figure 13.

Figure 16 compares the normal-incidence absorption coefficient spectrum predicted for the 60 mm thick Figure 3a design with absorption coefficient data for a 60 mm thick sample of Typha fiber.

The absorption coefficient data for the 60 mm thick Typha fiber sample above 1 kHz, with an average absorption coefficient value near 0.8, does not match the values predicted for the Figure 3a design. However, the absorption of the Typha fiber sample is higher between 500 Hz and 1 kHz. This results in a higher FOM for the Typha fiber sample (see Table 8) than that for the Figure 2a structure (6.430).

## 5. Conclusions

Similar or higher broadband sound absorption spectra than those measured and predicted for three relatively complex designs, based on resonant cavities, synthetic polymer foams, embedded arrays of horizontal plates in a porous layer, and parallel microperforated plates, can be achieved with relatively simple partition arrangements in a porous layer. The overall broadband absorption performance has been judged by using a Figure of Merit (FOM) with a target normal-incidence absorption coefficient of 0.8. The FOM takes into account not only the bandwidth at or above the target absorption but also the lowest frequency at which the target absorption is reached. The FOM calculated for the partitioned porous layers is comparable with or higher than those calculated for three more complicated metamaterial structures. Partition designs with additional components could offer further improvement [6], but the specific types considered demonstrate their potential. Since this potential has been demonstrated only through numerical and approximate analytical predictions, there is a need for manufacturing and testing of appropriate samples.

Another alternative that has been investigated is the use of porous materials made from natural sources with higher tortuosity values than those found for typical synthetic foams. Naturally sourced materials would have less environmental impact in both manufacturing and disposal than synthetic foams. On the other hand, naturally sourced materials have higher bulk densities than typical synthetic foams, which could mean significant additional structural loading, particularly if 60 mm thick layers were to be used. However, it would be possible to reduce the additional weight while preserving the sound absorption performance by using thinner layers with air gaps behind them.

## Figures and Tables

**Table 1:** Johnson–Champoux–Allard model (JCA) parameter values for two foams [1] and a ‘sponge’ [2].

Material	Porosity ϕ	Flow Resistivity (kPa s m^−2^)	Tortuosity $\alpha_{\infty }$	Viscous Characteristic Length Λ (μm)	Thermal Characteristic Length $\Lambda^{\prime }$ (μm)
melamine [1]	0.97	19.78	1.02	90.3	178.5
cavity [1]	0.95	11.66	1.07	78.2	155.3
sponge [2]	0.96	2.843	1.07	273	672

**Table 2:** FOM calculations for the designs in Figure 1a, Figure 2a and Figure 3a.

Design	$\alpha_{avg}$	$f_{r}$ Hz	∆f Hz	$\lambda_{max}$ m	L m	FOM
Figure 1a	0.875	9200	8600	0.429	0.031	10.992
Figure 2a	0.919	5355	4647	0.398	0.050	6.340
Figure 3a	0.928	5900	5200	0.429	0.060	5.846

**Table 3:** FOM calculations for type 2 and type 3 partitions in 31 mm thick ‘cavity’ foam.

Partition Type	$\alpha_{avg}$	$f_{r}$ Hz	∆f Hz	$\lambda_{max}$ m	FOM
Type 2	0.912	9400	8500	0.381	11.402
Type 3	0.956	9400	8400	0.343	9.457

**Table 4:** FOM calculations for a 50 mm thick layer of (unpartitioned) melamine foam and a 50 mm thick foam ‘sponge’ with type 3 partitions.

	$\alpha_{avg}$	$f_{r}$ Hz	∆f Hz	$\lambda_{max}$ m	FOM
Melamine foam	0.950	5300	4500	0.429	6.92
Type 3 partitions in ‘sponge’	0.920	5300	4450	0.404	6.23

**Table 5:** Parameter values for sugarcane bagasse and Typha fiber samples [11,12].

Material (Density, Thickness)	Porosity ϕ	Flow Resistivity (kPa s m^−2^)	Tortuosity $\alpha_{\infty }$	Viscous Characteristic Length Λ (μm)	Thermal Characteristic Length $\Lambda^{\prime }$ (μm)
Sugar cane bagasse (200 kg m^−3^, 30 mm)	0.836	6.120	1.7	54	211
Typha fiber (200 kg m^−3^, 30 mm)	0.857	23.972	2.5	90	120
Typha fiber (150 kg m^−3^, 50 mm)	0.920	15.26	2.8	85	150
Typha fiber (150 kg m^−3^, 50 mm)	0.920	15.78	1.5	70	380
Typha fiber (200 kg m^−3^, 50 mm)	0.892	19.77	2.8	120	180

**Table 6:** FOM calculated from Equation (10) and data for 30 mm thick hard-backed layers of samples made from Typha fiber and sugarcane bagasse.

	$\alpha_{avg}$	$f_{r}$ Hz	∆f Hz	$\lambda_{max}$ m	FOM
Typha fiber	0.8571	6100	5800	0.686	18.630
Sugarcane bagasse	0.8800	6100	5200	0.312	7.797

**Table 7:** FOM calculated from Equation (10) and data for 50 mm thick hard-backed samples made from Typha fiber.

Typha Sample Density; Binder %	$\alpha_{avg}$	$f_{r}$ Hz	∆f Hz	$\lambda_{max}$ m	FOM
150 kg m^−3^; 10	0.857	6200	5500	0.429	6.520
150 kg m^−3^; 7.5	0.827	6200	5670	0.544	8.235
200 kg m^−3^; 7.5	0.857	6200	5670	0.544	8.527

**Table 8:** FOM calculated from Equation (10) and data for a 60 mm-thick hard-backed sample made from Typha fiber.

Density; Binder %	$\alpha_{avg}$	$f_{r}$ Hz	∆f Hz	$\lambda_{max}$ m	FOM
200 kg m^−3^; 10	0.857	6200	5800	0.646	8.632

## Data Availability

The original contributions presented in this study are included in the article. Further inquiries can be directed at the corresponding author.
